# The relation between family socioeconomic status and depressive symptoms among children and adolescents in mainland China: a meta-analysis

**DOI:** 10.3389/fpubh.2023.1292411

**Published:** 2024-01-09

**Authors:** Yingnan Niu, Xiaolin Guo, He Cai, Liang Luo

**Affiliations:** ^1^Collaborative Innovation Center of Assessment for Basic Education Quality, Beijing Normal University, Beijing, China; ^2^Institute of Developmental Psychology, Beijing Normal University, Beijing, China; ^3^State Key Laboratory of Cognitive Neuroscience and Learning, Beijing Normal University, Beijing, China

**Keywords:** socioeconomic status, depressive symptoms, children, adolescents, mainland China, meta-analysis

## Abstract

Family socioeconomic status (SES) is widely believed to be associated with depressive symptoms in children and adolescents. The correlation between SES and depressive symptoms changes based on social culture and the economic development level. In China, which includes many children and adolescents, the magnitude of the relationship between SES and depressive symptoms and its potential moderators remains unclear. The current meta-analysis was conducted to determine the overall association between SES and depressive symptoms in children and adolescents in mainland China. We included 197 estimates in mainland China from 2000–2023. Among 147,613 children and adolescents aged 7–18 years, the results showed a weak but significant overall negative association between SES and depression (*r* = −0.076). Moderator testing showed that the composite SES indicator (*r* = −0.104) had a stronger association with depression than parental educational level (*r* = −0.065) and occupational status (*r* = −0.025) but not family income (*r* = −0.088). Additionally, the negative association between SES and depression became weaker over the past 20 years in China (*β* = 0.010). Furthermore, the magnitude of the relationship between SES and depression was stronger in West China (*r* = −0.094) than in Middle China (*r* = −0.065), but not East China (*r* = −0.075). These findings indicate that the relationship between SES and depression among children and adolescents in mainland China may vary based on social contexts. It is necessary to further explore the effect of these social factors and the underlying mechanisms.

## 1 Introduction

Depression is considered one of the major factors in the global disease burden (1). This disorder affects 2–8% of children and adolescents and peaks in adolescence (2). Depression in young people is associated with a series of negative outcomes, such as poor academic achievement, negative peer relationships and suicide (2). Therefore, exploring the factors associated with depression in children and adolescents is of great significance.

Many factors have been found to be related to children’s developmental outcomes (3). However, among these factors, lower family socioeconomic status (SES) is widely believed to be harmful to the development of children and adolescents. Therefore, as one of the adverse outcomes of child and adolescent development, depression is also influenced by the family socioeconomic status (4, 5). As the social causation hypothesis (6) notes, children and adolescents with lower SES are at a higher risk for psychopathology, including depression. The association between family SES and depression reflects the inequality in mental health. Specifically, children from lower-SES families may face a higher risk of depression due to the lack of necessary resources, while children from higher-SES families are at lower risk because they have access to more resources to help them overcome adversity and reduce depression. In China, with the rapid development of the economy, children and adolescents are facing increasingly serious mental health issues. For example, a study found that the detection rate of depression was 14.6% among primary school students in China, 24% among middle school students, and 28% among high school students, which suggests that the incidence of depression is increasing from childhood to adolescence (7). However, the high incidence of depression among children and adolescents may be related to the rapid development of Chinese society (8). China is an area of reform and development, and rapid economic development has led to increasing division between the rich and the poor, making social mobility more difficult and social stratification more prominent. As mentioned earlier, in today’s China, the impact of SES on depression in children and adolescents seems to be greater than ever before.

Therefore, based on the importance of SES for depression in children and adolescents in the rapidly developing context of China, numerous studies have explored the relationship between family SES and depression among children and adolescents in this context. Although most studies have found a negative relationship between family SES and children’s depression, findings on the magnitude of these relationships have been inconsistent (8, 9). This inconsistency may be due to the heterogeneity across studies, including the various measurements of family SES and depressive symptoms and the different characteristics of the study samples. For example, the equalization hypothesis suggests that the association between family SES and mental illness, including depression, is usually stronger in children than in adolescents (5), which demonstrates the “equalization” that occurs in adolescence (10). Similarly, there is agreement that parental educational level is more stable than other SES measurements in the assessment of the relationship between SES and depressive symptoms (11). In addition, the relationship between family SES and mental disorders, including depression, may be a function of gender (5).

Given the inconsistency among studies, a single study cannot fully reflect this relationship. Therefore, it is necessary to conduct a meta-analysis to reveal the overall magnitude of the association between family SES and depression among children and adolescents and to explore potential moderators that could be sources of the heterogeneity in previous studies. This analysis can reveal the association between SES and depression among children and adolescents in the context of rapid social development in China.

### 1.1 Rationale for the current meta-analysis

The unique correlation between family SES and depression in China, as mentioned earlier, deserves attention. However, previous meta-analyses have not specifically addressed this issue. To our knowledge, eight meta-analyses have explored the association between SES and depressive symptoms in children and adolescents (3, 4, 9, 12–15). Specifically, six studies have focused on children and adolescents: four of them examined the relationship between SES and developmental outcomes, including depression or internalizing behaviors, in Western countries, and one was conducted in mainland China. A recent meta-analysis conducted by Peverill et al. (14) revealed a small overall association of SES with children’s internalizing behavior (Hedge’s *g* = 0.22) in the United States. Additionally, Letourneau et al. (4) found a very weak but significant association between SES and internalizing behaviors (Hedges’ *g* = 0.08) in the United States. Furthermore, a meta-analysis conducted by Quon and McGrath (15) found that higher subjective SES was related to better mental health, including depression, in America, Europe, Asia and Australia, with a Fisher’s *Z* = 0.189. In the only study based on a Chinese sample (12), the findings indicated a moderate correlation between SES and depression (*r* = −0.15) among adolescents. Two studies examined the correlation of related factors, including family SES and depression, in Western countries. Specifically, Twenge and Nolen-Hoeksema (9) found no correlation between CDI scores and SES (*r* = 0.06 or below) in the United States and Canada, and Su et al. (3) found that lower SES (*OR* = 1.29) and maternal education (*OR* = 1.37) both increased the risk of depression in offspring in America and Europe. Furthermore, two studies focused on a wide range of age groups. Korous et al. (13) found a small but significant correlation (*r* = 0.04) between SES and internalizing behaviors among children and adolescents in the United States, while Zhang et al. (8) found a moderate negative correlation between SES and depression among children (*r* = −0.11) and adolescents (*r* = −0.14).

Based on the above analyses, it appears that no prior meta-analyses have specifically examined the distinct correlation between family SES and depression in children and adolescents in China. It is crucial to elucidate this unique relationship. First, identifying the overall magnitude of the association between family SES and depression in children and adolescents in the cultural context of China helps us understand the overall impact of SES on depression in Chinese children and adolescents which has not been clarified previously. Second, the magnitude of the correlation between SES and various mental health outcomes may vary. A previous study found that the relationship between SES and externalizing behavior was more robust than the relationship between SES and internalizing behavior (16). Focusing on the relationship between SES and general mental health may mask the unique relationship between SES and depression. Third, the moderating effects on the relations between SES and different mental health outcomes are also different. For example, previous findings revealed that the moderating effect of age may vary with specific mental health outcomes (17). In addition, potential bias may exist in the estimation of the magnitude of family SES and depression because insufficient sample sizes were included due to their extensive attention to multiple indicators (3, 4, 9, 13–16). Therefore, it is essential to conduct a meta-analysis to investigate the unique correlation between SES and depression in China along with its potential moderators.

### 1.2 Rationale for the focus in the current meta-analysis on the Chinese sample

First, as mentioned earlier, the rapid development of society in China has exacerbated the wealth gap and made social mobility more difficult, while the wealth gap has intensified the inequality of depression among children and adolescents. It seems that SES has an indelible impact on depression in children and adolescents in the context of the new era in China. However, the extent of the influence of SES on Chinese children and adolescents is still unknown because current research results on the relationship between SES and depression in Chinese children and adolescents are scattered and chaotic, making it impossible to derive a comprehensive result. Therefore, systematic summarization and organization, such as meta-analysis, are needed to clarify the strength of the relationship between SES and depression among Chinese children and adolescents and provide a theoretical and empirical basis for future research and interventions.

Second, we believe that the Chinese context provides a unique perspective on the socioeconomic disparity in children’s depression. Specifically, in the last 40 years of its reform and opening-up policy, China has achieved remarkable progress in different facets of development, including economy, society and culture, but it also faces some challenges, such as a higher divorce rate and income inequality (18). Social progress and social challenges may both affect the relationship between SES and depression among children and adolescents. However, it is unclear which aspects affect this relationship most. The contradictory development of Chinese society means that in recent decades, the development of Chinese society has achieved rapid progress in areas such as the economy, education, and culture, while at the same time, this progress has been accompanied by social issues such as increasing income inequality and uneven distribution of resources. This situation provides an excellent model to test whether social progress or social problems have a greater impact on the relationship between family socioeconomic status and depression among children and adolescents.

**Figure 1:**
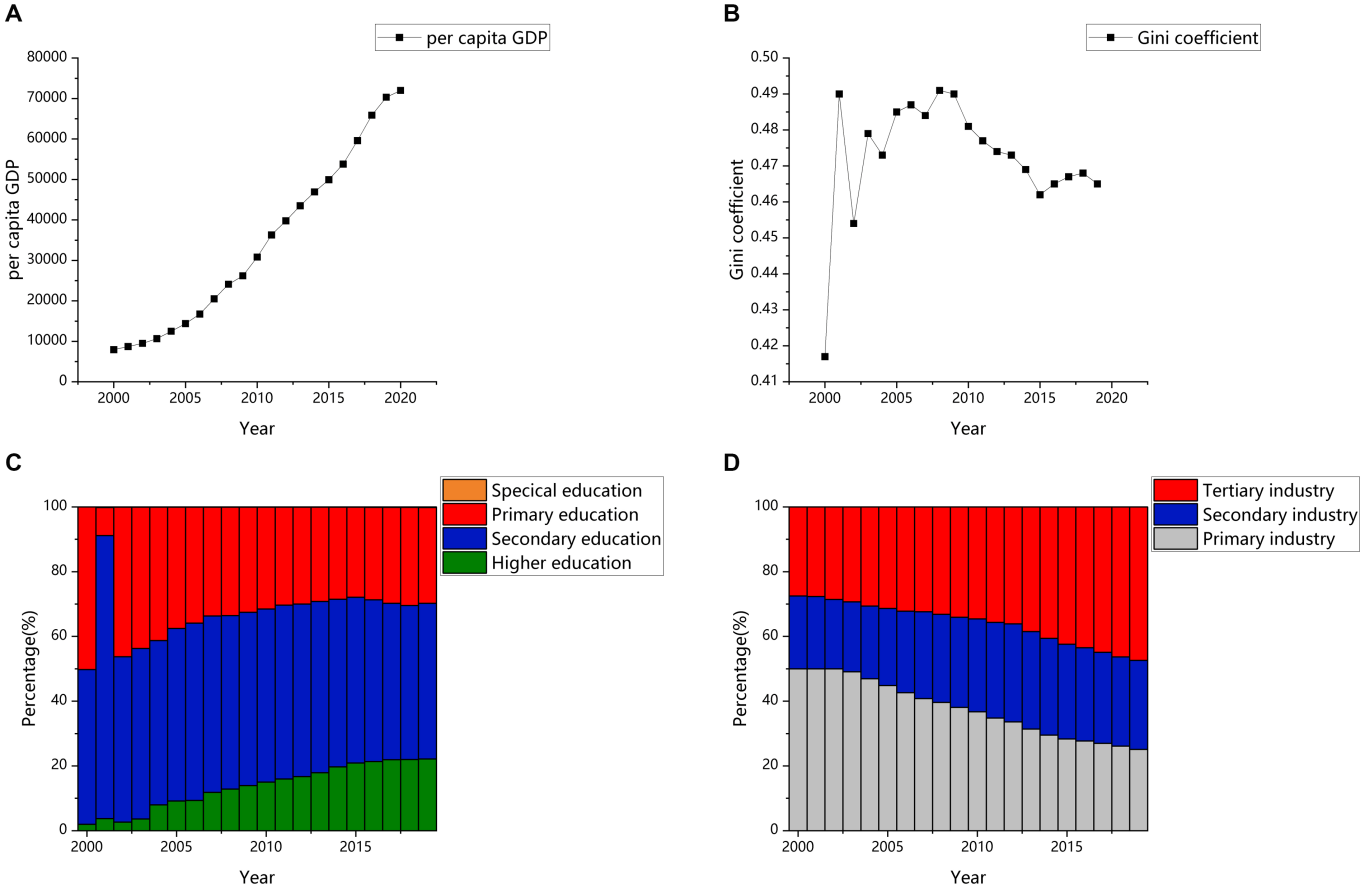
**(A)** *Per capita* GDP index in China. **(B)** Gini coefficient in China. **(C)** Distribution of academic qualifications among graduates in China. **(D)** Distribution of Chinese residents employed in three industries. Data source: **(A,C,D)** are from NBSC: 2000–2020 https://www.stats.gov.cn/sj/ndsj/). **(B)** is mainly from the relevant study results in China.

On the one hand, China has made great progress in the past 20 years. This social progress may reduce social inequality in depression. Specifically, the composition of SES has changed dramatically (19). With regard to income, the *per capita* GDP steadily increased from 7,942 yuan in 2000 to 72,000 yuan in 2020, an increase of 9 times (see Figure 1A). With regard to educational level, the proportion of the population receiving secondary education and higher education has been rising in the past 20 years (see Figure 1C). In addition, for occupation, the proportion of tertiary industry and secondary industry has increased year by year, while the opposite trend has been observed for primary industry in the past 20 years (see Figure 1D). Additionally, a set of measures was released by the Ministry of Education of China to improve mental health targets for children and adolescents, such as establishing psychological counseling rooms in schools, offering courses related to mental health, and establishing demonstration areas for mental health education. All these measures have been beneficial for reducing children’s depression. For example, in the past, only families with higher SES were able to access psychological counseling. However, due to the progress and development of Chinese society, children generally have opportunities to access mental health resources. Therefore, in today’s China, even children from low-SES families can access mental health resources, which allows children from families with different SES to receive timely help when facing the risk of depression, thus narrowing the gap in the prevalence of depression among children from families with different levels of SES. Therefore, progress in SES and mental health may contribute to the decrease in the social inequality of depression.

However, social progress is accompanied by many social problems, which may widen the social disparity in children’s depression. For example, there have been persistent income disparities in China in the past 20 years. The year 2000 was an important turning point, when the Gini coefficient exceeded 0.4 (0.4 indicates a large income gap) for the first time compared to the past decades. It has consistently remained above 0.45, indicating that China’s income gap has remained at a relatively high level since the beginning of the new century (see Figure 1B). This issue requires vigilance because this income disparity may strengthen the association between SES and well-being (20). For example, the high detection rate of depression in children from low-SES families may be due to a lack of resources, and resources related to depression may include not only the aforementioned mental health resources, but also economic resources, educational resources, and cultural resources. In today’s China, income inequality is increasing, and the gap in resources available to children from families with different SES is also widening. The widening of this resource gap will also lead to the widening of the gap in the incidence of depression among children from families with different levels of SES. Therefore, social problems such as higher income inequality in China could strengthen social inequality in depression.

As mentioned above, China’s great progress and social problems may result in two distinct developments in the relationship between SES and depression. Specifically, the popularization of mental health resources may narrow the gap in depression among children from families with different levels of SES, and widening income inequality may exacerbate the gap in depression among children from different SES families. It is unclear which aspect has a greater impact on the association between SES and depression. Therefore, the great changes in the Chinese context provide an excellent model to examine the relative importance of social progress and social challenges on social disparity in depression among children and adolescents. Thus, the current meta-analysis was conducted to reveal the developments that have occurred in the past 20 years.

In addition, China is characterized by a Confucian culture and collectivism. As the core of Confucian culture, family is regarded as an important part of individuals’ lives, so the influence of family on the individual seems more important in China. In addition, a previous study indicated that individuals from collectivist societies are more sensitive to stressful life events, such as having lower SES, and manifest more depression (21). On the other hand, collectivism also emphasizes the interdependence of individuals, social embeddedness, obligations, and loyalty to internal groups (such as families) (22). Therefore, in collectivist cultures, socioeconomically disadvantaged individuals may encounter social difficulties, but they often receive support and assistance from their families, which can alleviate the negative effects of their disadvantaged situation to some extent and ultimately reduce the risk of depression (8). However, the nature of the association between family SES and depression in children and adolescents remains unclear in the context of collectivist culture. Therefore, the exploration of this association could reveal the unique relationship between SES and depression among children and adolescents in China.

Notably, there has been one meta-analysis conducted using a Chinese sample (12). However, there are reasons to conduct a further meta-analysis that mainly focuses on the unique relationship between SES and depression among children and adolescents in mainland China: (1) an insufficient number of studies were included, and only 21 effect sizes were included for the SES-depression correlation, which may have biased the conclusions; (2) the samples for the SES-depression correlation mainly focused on adolescents and excluded children, which prevented an examination of changes in the social disparity in depression in the important period of the transition from childhood to adolescence (10); (3) the moderators were only used to examine the relationship between SES and mental health. As mentioned above, the moderating effect could be different for different outcome variables, such as age (17). Therefore, a moderator analysis needs to be conducted for the unique relationship between SES and depression; (4) Jia and Zhu (12) did not examine the association between different objective SES indicators and depression, and this association may vary with different objective SES indicators. Therefore, it is necessary to conduct a meta-analysis that includes sufficient samples to focus on the unique relationship between SES and depression and to explore its potential moderators based on Chinese children and adolescents.

Based on these analyses, a meta-analysis was conducted to examine the magnitude of the relation between family SES and depressive symptoms among children and adolescents in mainland China. Some moderators were examined as follows.

### 1.3 Potential moderators of the association between family SES and depressive symptoms

*Year:* As mentioned above, since the implementation of China’s reform and opening up policy, significant advancements have been achieved in the country; however, China has also been confronted with social challenges (18). The economic situation of children in poverty has continued to improve over the past 20 years in China because of the social progress. The Chinese government has promoted many policies aimed at providing economic support for poor children in rural areas to help them successfully complete their studies. For example, tuition and miscellaneous fees for poor children in rural areas are exempted, textbooks are provided free of charge, and living expenses for boarding students are subsidized. In addition, to improve the mental health of children, the Chinese government has implemented a range of policies, such as carrying out mental health education in schools, establishing psychological counseling rooms, and allocating necessary mental health education professionals. All of these changes may weaken the social disparity in depression among children and adolescents.

With regard to social problems, it is worth noting that income disparity remains at a relatively high level, and this disparity may strengthen the association between family SES and happiness (20). In addition, higher unemployment rates, crime rates and divorce rates have all been proven to be related to adolescents’ depression (18). Therefore, these social problems may strengthen the social disparity in depression among children and adolescents. Given the contradictory development of Chinese society, there seem to be two distinct possible developments in the association between family SES and depression across the past 20 years, namely, whether the association increased or decreased. Thus, how this relationship has changed in the past 20 years is an important question to be explored. In this study, a meta-analysis was conducted to explore the variation in this relationship over the years.

*Grade Level:* The association between family SES and depressive symptoms may differ across various stages of development. According to the “equalization hypothesis” (10), when adolescents reach puberty, they become more independent and gradually individualize from their parents. Thus, as adolescents grow older, the impact of family on their life decreases while the significance of school-related factors such as social connections with peers becomes more prominent. For example, as children age, they have fewer opportunities to interact with their families and more opportunities to interact with their schools and peers. As a result, the influence of the family, including family SES, on children should decrease. Therefore, the association between family socioeconomic status (SES) and depressive symptoms decreases during adolescence compared to early childhood, which is manifested as the “equalization” of depressive symptoms in adolescence. In this study, we aimed to examine this hypothesis based on different grade levels in the Chinese context.

*Economic Region:* The relationship between income and subjective well-being is affected by economic development; specifically, an increase in income does not improve happiness when the level of economic development is higher than a certain threshold, which is the famous “Easterlin paradox” (23). If money cannot buy happiness in a developed economy, can it reduce depression? It is reasonable to examine this question because negative affect and positive affect are two relatively independent aspects of well-being (24). Indeed, previous findings have shown that although money cannot buy happiness, it can reduce depression (25). Therefore, whether the Easterlin paradox exists in the relation between SES and depression needs to be further explored. Furthermore, previous studies of the Easterlin paradox have mainly focused on adults. However, SES seems to be more important for determining health in childhood than in adulthood because health status during adulthood is determined more by socioeconomic status during childhood. Specifically, according to the latency model, SES during early childhood has a lasting impact on adult health, even when considering SES during adulthood (26–28). These researchers argue that childhood is a crucial period during which children from lower-SES backgrounds may miss out on important developmental milestones, leading to negative health outcomes that persist into adulthood. Indeed, according to the critical period theory, childhood adversity determines depression in adulthood (29). For example, lower SES in childhood can lead to higher levels of depression in adulthood and cannot be improved even with social mobility in adulthood. This means that high socioeconomic status in adulthood cannot compensate for the harm that low socioeconomic status brings to depression in childhood, indicating that socioeconomic status seems more important in childhood than in adulthood. In the current study, economic region was used as the moderator to explore whether the Easterlin paradox exists in the relationship between family SES and children’s depression. In China, the economic regions are divided into three regions according to the level of economic development,^1^ namely, the East, Middle and West. The East is the most economically developed region, while the West is the least developed region, and the Middle region is between them. In 2020, the mean *per capita* disposable incomes in East, Middle and West China were 43,351 yuan, 27,118 yuan and 22,926 yuan, respectively.^2^ In this study, we examined whether this relationship among children and adolescents is moderated by economic regions.

*Gender:* Previous findings (30) have revealed that girls with lower parental education levels may have more severe depressive symptoms than boys. According to the diathesis-stress model (31), girls, who are often regarded as susceptible are more likely to suffer from adverse environments, such as lower SES, and thus show more depressive symptoms. Thus, girls with lower family SES may show more depressive symptoms than boys. This difference is supported by previous findings (32). The current meta-analysis seeks to examine whether the associations between family SES and depressive symptoms vary by gender among children and adolescents in mainland China.

*The Type of SES Assessment:* SES is usually divided into single indicators (parental educational level, occupational status and family income) and composite indicators. Previous evidence (13) consistently shows that single indicators have a stronger association with depression than composite SES indicators because composite SES indicators reduce the amount of available information in single indicators. For instance, Korous et al. (13) revealed that educational attainment and household income had a stronger association with internalizing behavior than overall SES in a population-based sample. There are also differences among single indicators; for example, parental educational level has consistently been found to be more stable than other single SES measurements in the assessment of the relation of SES with depressive symptoms, such as family income and parental occupation (11). In this study, we aimed to examine whether single indicators such as parental educational level are more informative than composite indicators in predicting depression among children and adolescents in mainland China.

*Type of Depressive Symptoms Assessment:* Different screening instruments and cutoff scores may lead to different prevalences of depressive symptoms (33); however, the differences are more likely to result from the methodological aspect than the substantive aspect (34). For example, a meta-analysis (35) based on a Chinese sample revealed that the prevalence estimates identified by the SDS (cutoff ≥ 40) were significantly higher than those identified by the SCL-90 (cutoff ≥ 2), CDI (cutoff ≥ 19) and CES-D (cutoff ≥ 2). Moreover, the CES-DC is mainly used in assessing children’s and adolescents’ depression, while the CES-D, PHQ-9 and SDS were primarily developed for adults (36). In addition, one study found that the correlation between the SDS and CES-D was smaller in Chinese samples (0.5974) than in Western countries (0.90) (37). The current meta-analysis included different types of instruments, such as the CDI, CES-D, SDS, and SCL-90, to explore whether the associations between SES and depressive symptoms among children and adolescents were moderated by different depressive symptom screening instruments.

## 2 Research questions

The current meta-analysis mainly addressed two questions.

*Question 1*: What is the overall magnitude of the association between family SES and depressive symptoms in mainland China among children and adolescents?

*Question 2*: What are the potential moderators of the association between family SES and depressive symptoms in mainland China among children and adolescents?

## 3 Methods

### 3.1 Screening criteria for studies

The inclusion criteria were as follows:

1. The research focused on the relationship between family SES and depressive symptoms.
2. Research in which sufficient statistical details were reported to calculate the correlation between family SES and depressive symptoms, such as the *r*, means (*M*), sample size (*N*) and standard deviations (*SD*).
3. Typically developing students from primary school to high school (aged 7–18) in mainland China were included in the research sample.
4. The study included published and unpublished articles written in Chinese or English.

The exclusion criteria were as follows:

1. The study contained significant errors or insufficient information.
2. The study included abnormal samples (such as children with disease).
3. The study included kindergarten or college students who could not be distinguished from the target sample.

### 3.2 Literature search and selection

**Figure 2:**
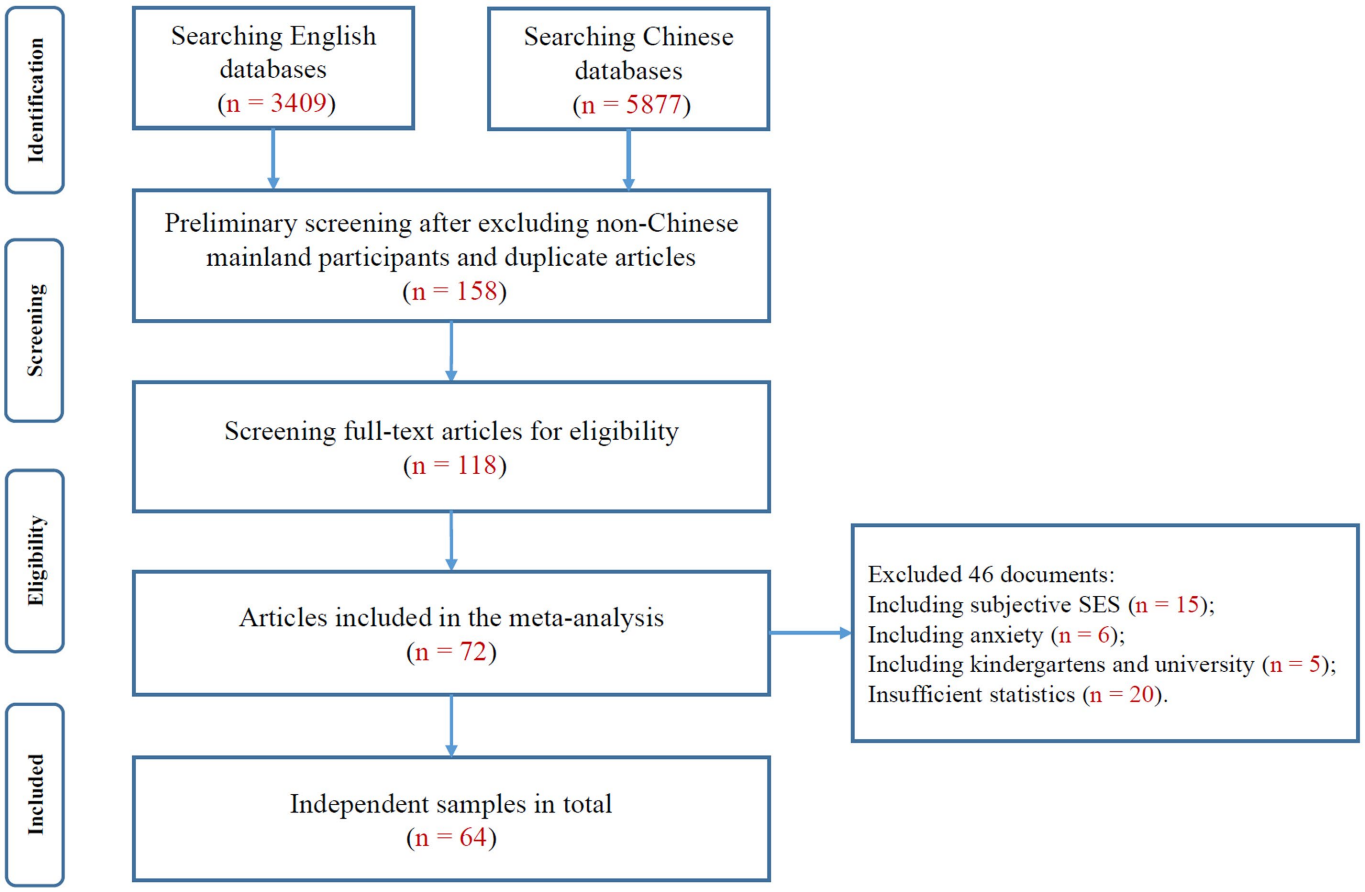
Flowchart of studies conducted from 2000 to 2023.

To identify relevant research on the relationship between family SES and depression, the following databases were searched electronically. For English research, the following databases were used: *Web of Science*, *PsycINFO*, *PubMed*, and *EBSCO-MEDLINE*. For Chinese research, the following databases were used: the CNKI database, Wanfang Database and VIP database. The following keywords were used: specifically, occupation* (“职业”) OR *social class (“社会阶层”)* OR education* (“学历/受教育水平”) OR *poverty (“贫穷”)* OR *social status (“社会地位”)* OR *socioeconomic status (SES, “社会经济地位”)* OR *income (“收入”)* were used for SES. For depression, we used the terms internalizing behavio* (“内化行为”) OR depress* (“抑郁”) OR *mental health (“心理健康”)* OR *mental disorder (“心理障碍”)* OR *emotion (“情绪”)*. The retrieval deadline was November 30, 2023. A total of 9,286 studies (3,409 in English databases and 5,877 in Chinese databases) were preliminarily identified according to their relevance to our topic. After studies with non-Chinese mainland participants and duplicate articles (*n* = 9,128) were excluded, 158 studies remained. We then read the full text carefully for eligibility, and 118 studies remained. According to the above screening criteria, 46 studies that did not meet the inclusion criteria (including subjective SES (*n* = 15), anxiety (*n* = 6), kindergarten and university education (*n* = 5), and insufficient statistics (*n* = 20)) were excluded. The current meta-analysis included 72 studies (54 Chinese and 18 English). The time span was from 2000 to 2023. We also searched the reference lists of related studies, such as meta-analyses, to ensure that relevant research was not omitted. If full-text information could not be obtained, we asked the author to provide us with the original text by e-mail (see Figure 2 for the flow chart).

The current meta-analysis ultimately included 72 studies (54 Chinese and 18 English) that yielded 64 independent samples and 197 effect sizes and included 147,613 participants from January 2000 to November 2023. The studies included in the current meta-analysis were categorized into two types: unpublished journal research (63 effect sizes) and published journal research [Chinese Social Sciences Citation Index (CSSCI), Social Sciences Citation Index (SSCI), or other journals, 134 effect sizes; for the references of the research data, see Appendix A].

### 3.3 Coding procedure

A set of data extraction tools (19) was used to collect relevant information from the original studies. The following characteristics were coded: (*a*) sample cohort year, for which we used the accurate sampling year if it was provided or the value (the publishing year minus 2) if it was not provided (19); (*b*) type of SES assessment (family income, education, occupation, composite SES index); (*c*) type of depressive symptom assessment (CDI, CES-D, SCL-90, SDS, and other measures), where other measures refer to those that cannot be classified as a separate category; (*d*) sample size *N* and all the effect sizes (*r*, *F*, *t*, *χ*^2^, *β*, *OR*); and (*e*) gender ratio (because most studies did not provide independent gender samples, we used the gender ratio as a continuous variable to examine its moderating effect, with a higher score indicating a higher proportion of boys); (*f*) grade level (primary school, middle school, high school); and (*g*) economic region (Eastern region, Central region, Western region). Additionally, we coded the study quality (according to 10 criteria, see Appendix B), publication status (unpublished vs. published) and calculation of depressive symptoms (continuous vs. categorical) as the covariates.

Each independent sample was uniquely encoded. Specifically, if a sample contained multiple effects or if multiple findings were drawn from the same sample, we coded it as the same ID. To reduce coding bias, two trained researchers first coded all studies independently and then resolved their inconsistencies through discussion (final interrater agreement > 90%).

### 3.4 Statistical strategies for the effect size

In the current study, the correlation coefficient *r* between family SES and depressive symptoms was used as the effect size because most previous meta-analyses have reported correlations. However, studies that did not provide the correlation, but gave sufficient information to compute the *r* value, such as *M*, *SD*, *t*, *χ*^2^, *F*, and sample *N,* were also included in the current study (38) (for details, see Appendix B).

All of the *r* values were converted into Fisher’s *Z* score [Eq. (B.2); see Appendix B], and the variance of each effect size was calculated after the correlation was obtained [Eq. (B.3); see Appendix B]. Fisher’s *Z* score was used for the whole statistical analysis and was subsequently converted back to the correlation coefficient [Eq. (B.4); see Appendix B] (39).

### 3.5 Analytical strategies

In the current study, we used analytical strategies that were used in a previous meta-analysis (19). Specifically, we included research that contained multiple effect sizes in one independent sample to consider as many eligible effect sizes as possible. However, the traditional meta-analytic approach, which relies on separate samples of subjects, may be unsuitable for analyzing numerous effect sizes that exist within clusters (40). Therefore, the random effects robust standard error estimation technique (41) was used to explain the within-cluster “multiple-outcome” dependencies because it could be used to analyze the clustered data. This method incorporates the correlation between different effect sizes from the same sample into the analysis and uses it to correct the standard error of the study. The method involves calculating the average correlation (*ρ*) among all effect size pairs within a cluster and determining the between-study sampling variance estimate, *τ*^2^. In our analysis, we set the value of *ρ* = 0.8 for *τ*^2^ estimation, and sensitivity analyses indicated that the findings remained consistent across varying values of *ρ* (41).

Weighted and random effects meta-regression models (41) were used to analyze the moderation effects. All moderators and covariates were incorporated into a single model to exclude the potential confounding effects of other moderators (19). This approach allowed us to estimate the unique effect of each moderator. In addition, we ran an independent model for the moderator “grade” because it only provided a partial of the independent sample (*n* = 27, *k* = 58). Prior to inclusion in the model, all categorical moderators were dummy coded (42). Additionally, any studies with missing data for the moderating analysis were excluded.

### 3.6 Publication bias

**Figure 3:**
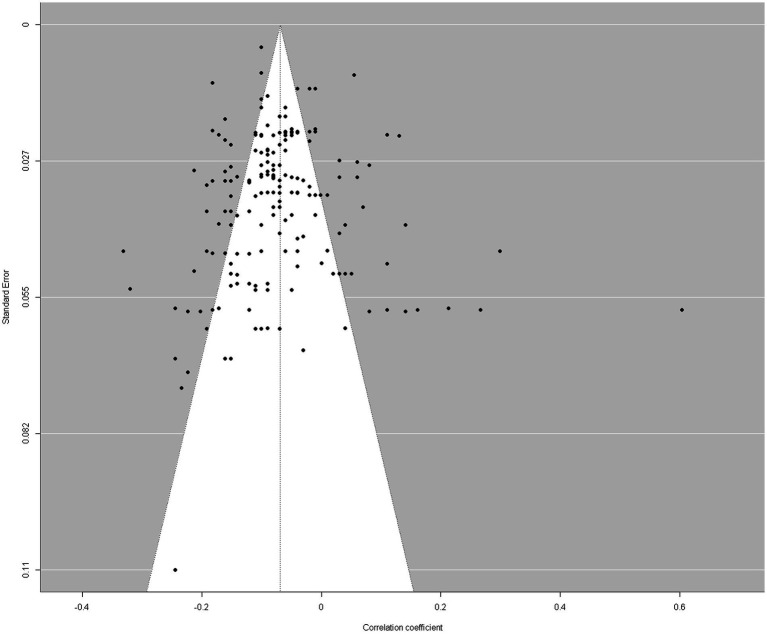
Funnel plot.

Publication bias refers to the concept that significant findings may be easier to accept or publish than nonsignificant findings (43). In this study, we used two methods to assess publication bias (19). The first method was Egger’s test (44). The results showed that there was no publication bias (*Z* = −0.09, *p* > 0.05; see Figure 3 for the funnel plot, the funnel plot shows that the effect sizes are concentrated at the top of the graph and symmetrically distributed on both sides of the total effect, indicating that there is no publication bias). The second method was a comparison of whether there was a significant difference between published studies and unpublished studies. Studies that had been published underwent peer review, while dissertations did not. Since peer-reviewed studies tend to report more significant results (19), the absence of significant differences between the published studies and unpublished studies would suggest that there was no bias in publication. According to the results, there was no discernible distinction between them (*β* = −0.056, *p* > 0.05; see Table 1, Model 1), suggesting no indication of publication bias.

**Table 1:** Moderating effects model summaries.

Model 1					
Interaction model	*β*	*SE*	*t*	95% CI	*p* value
**Year (continuous)**	**0.010**	**0.004**	**2.624**	**[0.002, 0.018]**	0.017*
Gender ratio (continuous)	−0.003	0.049	−0.063	[−0.110, 0.104]	0.951
Type of SES measure					
Occupation vs. education	0.066	0.042	1.569	[−0.023, 0.154]	0.135
Family income vs. education	−0.031	0.030	−1.061	[−0.093, 0.030]	0.299
**Composite SES vs. education**	**−0.075**	**0.032**	**−2.339**	**[−0.142, −0.008]**	0.030*
Family income vs. occupation	−0.097	0.049	−1.970	[−0.199, 0.005]	0.062
**Composite SES vs. occupation**	**−0.141**	**0.056**	**−2.498**	**[−0.258, −0.023]**	0.021*
Composite SES vs. family income	−0.044	0.040	−1.094	[−0.127, 0.040]	0.287
Type of depression measure					
CES-D vs. CDI	−0.017	0.030	−0.585	[−0.079, 0.044]	0.564
SCL-90 vs. CDI	0.043	0.045	0.939	[−0.053, 0.138]	0.360
SDS vs. CDI	−0.009	0.032	−0.274	[−0.080, 0.063]	0.790
Others vs. CDI	−0.069	0.041	−1.681	[−0.157, 0.020]	0.119
SCL-90 vs. CES-D	0.061	0.051	1.203	[−0.046, 0.168]	0.244
SDS vs. CES-D	0.011	0.030	0.362	[−0.057, 0.078]	0.726
Others vs. CES-D	−0.050	0.032	−1.548	[−0.121, 0.022]	0.154
SDS vs. SCL-90	−0.043	0.045	−0.962	[−0.142, 0.056]	0.357
Others vs. SCL-90	−0.103	0.057	−1.795	[−0.225, 0.020]	0.095
Others vs. SDS	−0.055	0.040	−1.382	[−0.141, 0.032]	0.192
Economic region					
Middle region vs. East region	0.033	0.024	1.399	[−0.018, 0.085]	0.184
West region vs. East region	−0.054	0.040	−1.352	[−0.141, 0.033]	0.201
**West region vs. Middle region**	**−0.088**	**0.039**	**−2.271**	**[−0.170, −0.005]**	0.038*
Publication status					
Published vs. unpublished	−0.056	0.030	−1.853	[−0.121, 0.009]	0.087
Calculation					
Continuous vs. categorial	0.026	0.025	1.023	[−0.028, 0.079]	0.322
Study quality	−0.002	0.004	−0.529	[−0.011, 0.006]	0.603
Model 2					
Grade level					
Middle school vs. primary school	0.079	0.082	0.960	[−0.116, 0.273]	0.370
High school vs. primary school	0.093	0.089	1.037	[−0.115, 0.300]	0.332
High school vs. middle school	0.014	0.074	0.189	[−0.165, 0.193]	0.856

Model 1: All moderators except grade level were entered into a single model. The second group is the reference group (e.g., in CES-D vs. CDI, CDI is the reference group). Gender refers to the ratio of males to females. The τ^2^ is 0.007 in the moderating effects Model 1 and 0.013 in the moderating effects Model 2. The variables in bold are the significant moderators. Model 2: The results of the moderating effect of grade level in another independent model controls for other moderators and covariates.

## 4 Results

### 4.1 Heterogeneity test

The heterogeneity testing results indicated that *Q* (*df* = 196) = 1669.37, *p* < 0.001, *I*^2^ = 94.24%, which exceeded the 75% rule proposed by Higgins et al. (45). This result indicated significant heterogeneity among studies, so the random-effects model needed to be conducted in the current study. In addition, these results suggest that the differences in research results may be influenced by some research characteristics. It was necessary to explore the moderating variables that affect the relationship between family SES and depressive symptoms among children and adolescents.

### 4.2 Overall effects

The overall effect (*k* = 197) was −0.076 with a 95% confidence interval [−0.098, −0.055], indicating that there was a weak negative association between family SES and depressive symptoms among mainland Chinese children and adolescents (see Table 2). The sensitivity analysis (leave-one-out analyses) suggested that after excluding any single effect size, the estimated result was stable at −0.07, 95%CI [− 0.08, − 0.05], *I*^2^ = 88%, indicating a stable negative correlation between socioeconomic status and depressive symptoms.

**Table 2:** Bivariate model summaries.

Model	*k*	*r*	95% CI of *r*	*τ* ^2^
Mean correlation	197	−0.076	[−0.098, −0.055]	0.006
Calculation				
Continuous	155	−0.075	[−0.103, −0.047]	0.007
Categorical	42	−0.076	[−0.109, −0.044]	0.004
Publication status				
Published	134	−0.086	[−0.107, −0.065]	0.005
Unpublished	63	−0.053	[−0.112, 0.006]	0.012
Grade level				
Primary school	11	−0.089	[−0.171, −0.006]	0.007
Middle school	34	−0.065	[−0.109, −0.020]	0.009
High school	35	−0.040	[−0.117, 0.038]	0.011
Economic region				
East region	112	−0.075	[−0.112, −0.038]	0.010
Middle region	41	−0.065	[−0.101, −0.029]	0.004
West region	17	−0.094	[−0.156, −0.032]	0.003
Type of SES measure				
Education	94	−0.065	[−0.087, −0.043]	0.005
Occupation	43	−0.025	[−0.098, 0.048]	0.014
Family income	30	−0.088	[−0.130, −0.045]	0.005
Composite SES	30	−0.104	[−0.142, −0.067]	0.005
Type of depression measure				
CDI	28	−0.097	[−0.139, −0.055]	0.005
CES-D	65	−0.072	[−0.100, −0.044]	0.003
SCL-90	32	−0.039	[−0.143, 0.066]	0.016
SDS	49	−0.088	[−0.144, −0.033]	0.004
Others	23	−0.094	[−0.164, −0.025]	0.013

k, number of effect sizes; CI, confidence interval, τ^2^, between-study sampling variance.

### 4.3 Moderating effects

We explored the potential moderating effects of year, gender ratio, economic region, grade level, type of assessment of SES, and type of assessment of depressive symptoms on the relation between family SES and depressive symptoms and conducted a subgroup analysis for each categorical moderator. After entering all moderators and covariates into one model simultaneously, we obtained the following results (see Table 1).

**Figure 4:**
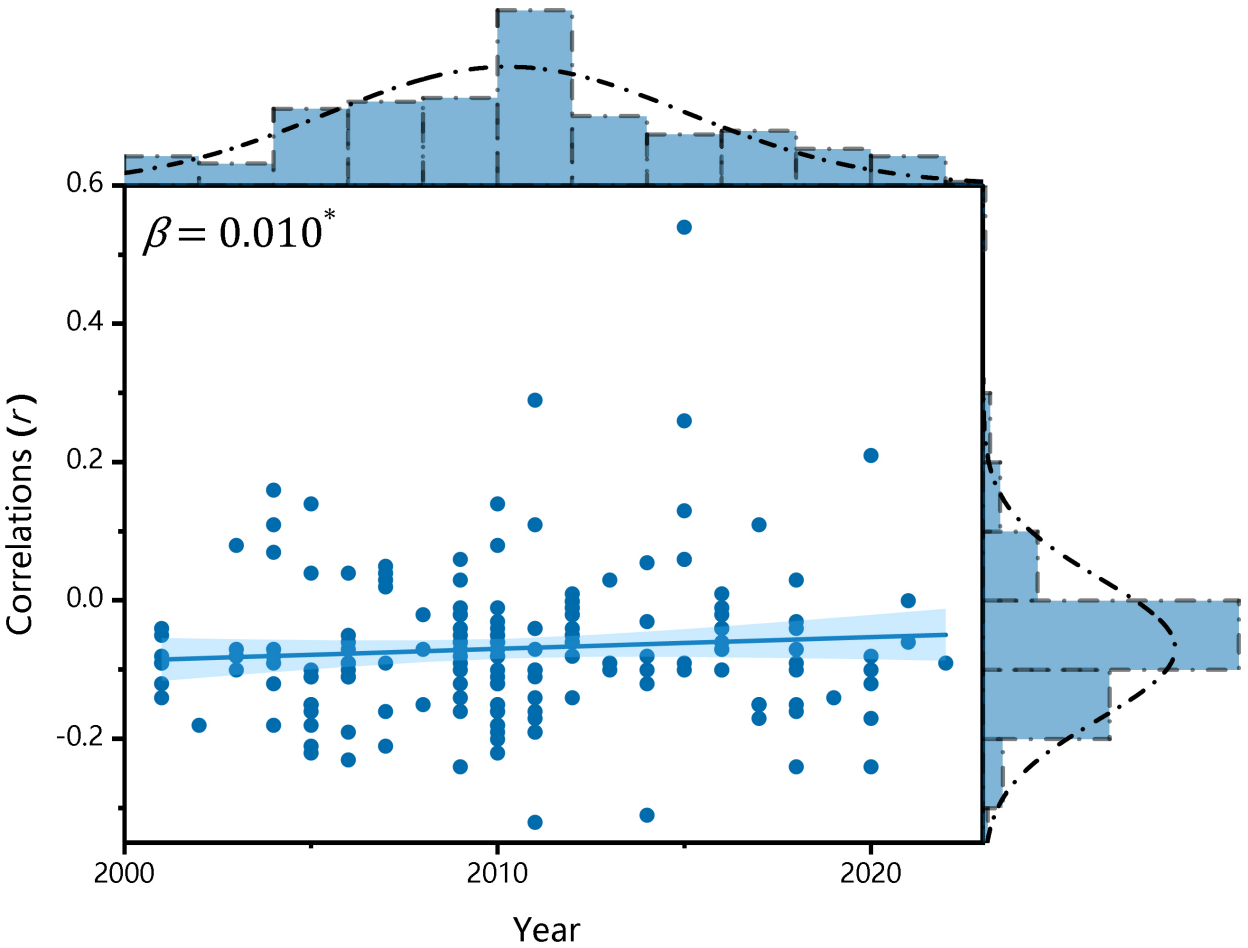
Variation in correlations between family SES and depressive symptoms over time.

*Year:* The current study included research conducted from 2000–2023. It is crucial to understand whether the association between family SES and depressive symptoms changed over time. The results showed that after controlling for confounding variables, there was a significant moderating effect of year (*β* = 0.010, *p* < 0.05), indicating that in the past two decades, there has been a decrease in the correlation between family SES and the prevalence of depressive symptoms in children and adolescents (see Table 1, Model 1; Figure 4).

*Gender:* We investigated how the gender ratio influenced the correlation between family SES and depressive symptoms. Our findings revealed that after adjusting for confounding variables, the gender ratio did not have a significant moderating effect (*β* = − 0.003, *p* > 0.05). This suggests that the relationship between family SES and depressive symptoms is similar for both male and female students (see Table 1, Model 1).

**Figure 5:**
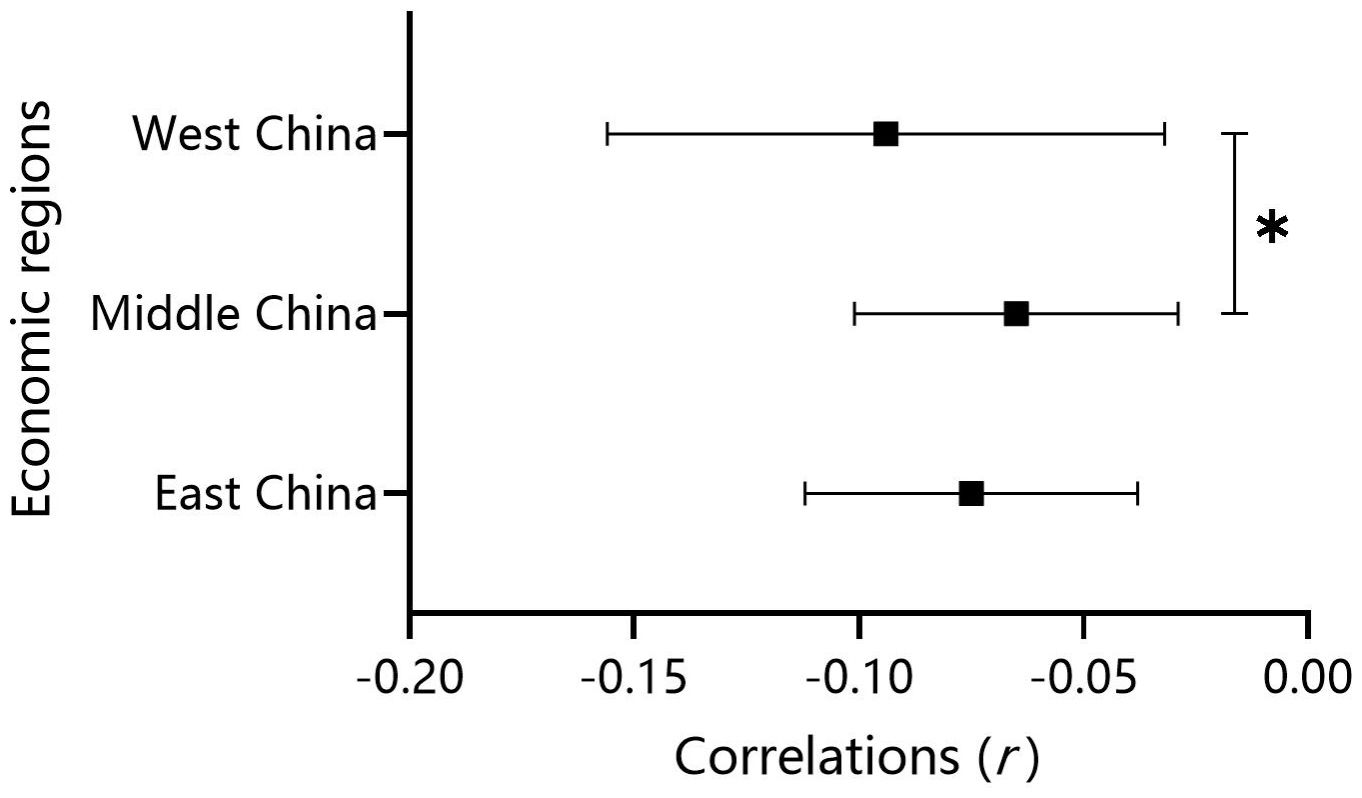
Effect size comparisons for the economic regions.

*Economic Region:* When analyzing the moderating effect of economic region, 112 correlations for the Eastern region, 41 correlations for the Middle region, and 17 correlations for the Western region were examined and considered in the final analysis. The average relation between family SES and depressive symptoms for each region was significant: East China, *r* = − 0.075, 95%CI [− 0.112, − 0.038]; Middle China, *r* = − 0.065, 95%CI [− 0.101, − 0.029]; West China, *r* = − 0.094, 95%CI [− 0.156, − 0.032] (see Table 2). After controlling the confounding variables, there was a significant difference between the Middle and the West (*β* = − 0.088, *p* < 0.05), while no differences were found in the other comparisons (see Table 1, Model 1, Figure 5).

*Grade Level:* As mentioned above, an independent model for grade level was developed and controlled for other moderators and covariates (see Table 1, Model 2). There were 11 correlations for primary school students, while for middle school students and high school students, 34 correlations and 35 correlations were obtained in the final analysis. The mean correlation between family SES and depression was not significant only for high school students: primary school, *r* = −0.089, 95%CI [−0.171, −0.006]; middle school students, *r* = −0.065, 95%CI [−0.109, −0.020]; high school students, *r* = −0.040, 95%CI [−0.117, 0.038] (see Table 2). After controlling for the confounding variables, no significant differences were found in any of the comparisons.

**Figure 6:**
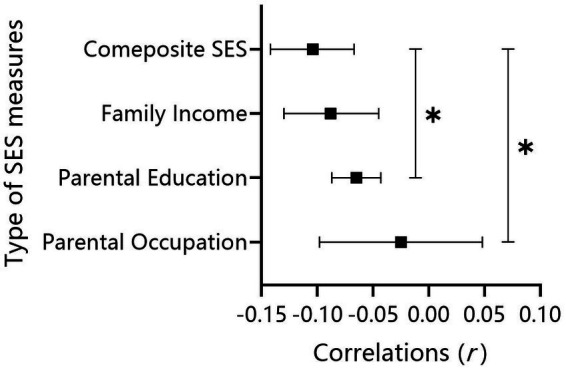
Effect size comparisons for the type of SES measures.

*Type of SES Measure:* In the analysis of the moderating effect of specific SES measures, a total of 94 associations for parental education, 43 associations for parental occupation, 30 associations for family income, and 30 associations for composite SES were examined and considered in the final analysis. The mean correlation between SES and depressive symptoms is only not significant in terms of parental occupation (see Table 2): parental education, *r* = −0.065, 95%CI [−0.087, −0.043]; parental occupation, *r* = −0.025, 95%CI [−0.098, 0.048]; family income, *r* = −0.088, 95%CI [−0.130, −0.045]; composite SES, *r* = −0.104, 95%CI [−0.142, −0.067]. After controlling for the confounding variables (see Table 1, Model 1), the correlations for parental education (*β* = −0.075, *p* < 0.05) and parental occupation (*β* = −0.141, *p* < 0.05) were significantly weaker than those for the composite SES. There were no significant differences among the other comparisons (see Figure 6).

*Type of Measurement of Depressive Symptoms:* When analyzing the moderating effect of specific measurements of depressive symptoms, a total of 28 associations for the CDI, 65 associations for the CES-D, 32 associations for the SCL-90, 49 associations for the SDS and 23 associations for other measures were examined and considered in the final analysis. The mean correlation between SES and depressive symptoms is only not significant in SCL-90 (see Table 2): CDI, *r* = −0.097, 95%CI [−0.139, −0.055]; CES-D, *r* = −0.072, 95%CI [−0.100, −0.044]; SCL-90, *r* = −0.039, 95%CI [−0.143, 0.066]; SDS, *r* = −0.088, 95%CI [−0.144, −0.033]; and other measures, *r* = −0.094, 95%CI [−0.164, −0.025]. After controlling for the confounding variables (see Table 1, Model 1), no significant differences were found in any of the comparisons.

## 5 Discussion

In this study, a meta-analysis was conducted to reveal the overall association between family SES and depressive symptoms among children and adolescents based on a Chinese sample and to examine how this relationship was moderated by potential moderators. We found a weak but significant negative correlation between family SES and depressive symptoms among children and adolescents in mainland China. In addition, we found significant moderating effects of years, economic regions and type of SES measures on the relationships between family SES and depressive symptoms.

### 5.1 Relationship between SES and depressive symptoms

Our results revealed that there was a weak but significant negative relationship between family SES and depressive symptoms among children and adolescents in mainland China (*r* = −0.076). While Jia and Zhu (12) found a moderate correlation between SES and depression among adolescents in mainland China (*r* = −0.15), this inconsistency can mainly be attributed to different target groups (middle school, university vs. primary school, middle school) and different SES indicators (subjective SES, objective SES vs. income, education, occupation). The weak but significant correlation revealed in the current study is consistent with the findings of Su et al. (3) for the Americas and Europe but is inconsistent with the findings of Twenge and Nolen-Hoeksema (9) for the United States and Canada. However, on the whole, the findings of the current study and previous studies (3, 9) revealed the similar result of a null-to-weak correlation between SES and depression among children and adolescents, indicating that a weak correlation is common across these countries. More meta-analyses based on other countries are needed for comparison to examine this universality.

### 5.2 Analysis of moderating effects

*Year:* In this study, we aimed to examine the trend of the association between family SES and children’s depression in the past 20 years. The current study found that the magnitude of the negative correlation between family SES and depressive symptoms among children and adolescents declined over the years, which supports the hypothesis that social progress is more closely related to this relationship. This is congruent with previous findings (12). Social progress may reduce the risk of children’s depression from the lower extreme of the social ladder. This can mainly be attributed to the policies issued by the Chinese government, such as poverty alleviation policies and mental health policies.

However, there is a compelling explanation for the declining trend: the decrease in social inequality is associated with the deterioration in depression of the whole spectrum on the social ladder. In China, educational competition in basic education has become increasingly fierce in recent years. Therefore, all children, even those with higher SES, may face similar pressures so that there is no social disparity in the occurrence of depression. Although we hoped that only the first situation would be true, we must admit that both situations may coexist.

*Type of SES Measure:* In contrast to previous findings (13), the current study found that the composite SES indicator was more likely to have a stronger or at least equal predictive effect on depression compared to single indicators. There were no differences among single indicators in China, indicating that the synthesis of each indicator was indispensable for interpreting the depressive symptoms of Chinese children and adolescents. The result suggests that when interpreting the depression of Chinese children and adolescents, it is not sufficient to consider only parental educational level, as indicated by previous findings (11). It is necessary to take family income and parental occupation into consideration simultaneously. According to need theory (46), in China, which is a typical developing country, income is a very important influencing factor for children’s mental health. Additionally, although no separate contribution of occupation was found in this study, it increased the informative power of the composite SES indicator because it is inseparable from income and education (11). Therefore, the current study reveals the differences between this study and previous studies of different SES indicators in predicting depression in children and adolescents. These results provide a research basis for targeted interventions for depression in children and adolescents in Chinese cultural backgrounds.

*Economic Region:* In the current study, we found that the correlation between family SES and children’s depression in the Middle region was significantly weaker than in the West. No significant differences were found between the Eastern and Middle regions or the Western region, which partially supports our hypothesis. The difference between the West and Middle regions is easy to understand. According to need theory (46), the material resources produced by objective SES are more important to children’s mental health in poor areas than in wealthier areas, leading to a stronger association between family SES and depression in the West than in the Middle region. However, with regard to the unexpected result in the East, a possible explanation could be that migrant children in the current study were all located in the East. According to status-anxiety theory (47), social inequality has a disproportionally deleterious impact on individuals with lower status. Migrant children are usually at the bottom of society and often suffer from an unfair distribution of social resources, resulting in more severe depressive symptoms and thus demonstrating no relative equalization in the East. This result is supported by previous findings indicating that migrants may have more mental health problems in developed cities (48).

*Gender:* In this study, we did not find gender differences in the relationship between family SES and depressive symptoms among children and adolescents. This is congruent with previous findings (30) but inconsistent with other findings (32). According to the stress-buffering hypothesis (49), social support may play an important protective role in the relationship between lower family SES and children’s depression. Although females are more sensitive to adverse environments, they are also likely to acquire more social support than males (50). Although girls may be more easily affected by adversity, they often receive more help than boys when facing the same difficulties. This may offset the harmful effect of lower social position and lead to a lack of gender differences in the relationship between family SES and depression.

*Grade Level:* With regard to the moderating effect of grade level, we found no variation in the strength of the relationship across different grade levels, which did not provide evidence for the equalization hypothesis (10). This finding supported the childhood-adolescent persistent model proposed by Chen et al. (17), which suggests that socioeconomic disparity in depression is established in early childhood and remains constant during the transition from childhood to adolescence in China. In other words, depression or, more broadly speaking, developmental outcomes, is already determined by the family in early life, and the differences in these family-induced developmental outcomes remain stable as children transition into adolescence. This result is of great significance and indicates that in China, early-life SES is very important because it leads to a constant social disparity in depression during the transition from childhood to adolescence. This means that the social support or other benefits produced by schools in later life cannot reduce the adverse effects of families in early life. Future research and interventions should aim to reduce social inequality in early life.

*Type of Depression Measure:* Finally, for the type of depression measure, we found no differences among all the comparisons, indicating that different measurements of depression did not influence the relationship between SES and children’s depressive symptoms in China. Additionally, since this question mostly involved the problem of measurement rather than the substantia of depression (34), we found no differences across different measurements. Therefore, we will not discuss this problem further.

## 6 Limitations

There are some limitations in the current meta-analysis. First, cross-sectional correlation studies were included in the current study, so a causal relationship could not be determined. Future studies could include more longitudinal studies to draw causal conclusions about the relationship between family SES and children’s depression. Second, due to limited/missing information, we did not include subjective SES in the current study. However, previous studies have shown that subjective SES seems to be a more predictive indicator than objective SES in the association with children’s depression (51). Future studies could include subjective SES as an important indicator to explore whether there is a stronger association between subjective SES and children’s depression than objective SES and children’s depression in China. Third, due to limited/missing information, some important moderators could not be explored, such as migrant status, income inequality and density, which warrants further investigation. Finally, since the current study used only meta-analysis to investigate the relationship between SES and depressive symptoms among children and adolescents in mainland China, the conclusions have certain limitations for extension to other samples. Therefore, more international samples will be included in future studies to compare with Chinese samples, and more research methods will be used to explore the relationship between SES and depression.

## 7 Conclusion

This is the first meta-analysis to focus mainly on children and adolescents. It examined the unique correlation and the potential moderators between family SES and depressive symptoms in children and adolescents in mainland China, which have not been clarified before. For the main relationship between SES and depression, we found a weak negative association between family SES and children’s depressive symptoms, reflecting a higher prevalence of depression in children with lower SES. In addition, for the moderator analysis, some important moderators were examined for this association. Specifically, year, economic region and type of SES measure significantly moderated the relationship between SES and depressive symptoms. For the year, we found that the strength of the association between SES and depressive symptoms declined in the past 20 years. For the economic region, the association was stronger in West China than in Central China but not East China. In addition, for the type of SES measure, the composite SES indicator seemed to be a more important indicator of children’s depression than single indicators because there was a stronger correlation between family SES and depressive symptoms for composite SES than parental education and parental occupation, but not family income. However, we did not find a moderating effect of gender, grade level or the type of depressive symptoms. Further studies are needed to clarify the underlying mechanisms between social factors and this relationship.

## Data availability statement

The raw data supporting the conclusions of this article will be made available by the authors, without undue reservation.

## Author contributions

YN: Conceptualization, Data curation, Formal analysis, Methodology, Visualization, Writing – original draft, Investigation, Project administration, Resources, Software, Validation, Writing-review & editing. XG: Supervision, Writing – review & editing. HC: Writing – review & editing. LL: Conceptualization, Funding acquisition, Supervision, Writing – review & editing.

## Footnotes

^1^During the period of China’s Seventh Five Year Plan, the whole country was divided into three economic zones: the East, the Middle and the West.

^2^China Statistical Yearbook 2021: https://www.stats.gov.cn/sj/ndsj/2021/indexch.htm
