# Supplementary material for: The relation between family socioeconomic status and depressive symptoms among children and adolescents in mainland China: a meta-analysis
Source: Front Public Health. 2024 Jan 9;11:1292411. doi: 10.3389/fpubh.2023.1292411 (PMC10803464; doi:10.3389/fpubh.2023.1292411)
Supplement: Supplementary file 1 [file Data_Sheet_1.pdf]

Supplementary Material

1 Appendix A – Data References (Literature included in the meta-analysis (72 studies))

1. Bai LY. Family environment of the ethnic group of she and it's impact on the mental health of their children of junior and high school. Master dissertation, Fujian Normal University (2003).
2. Cao YM, Lin XN, Chen L, Ji LQ, Zhang WX. The catechol-O-methyltransferase and dopamine transporter genes moderated the impact of peer relationships on adolescent depressive symptoms: A gene–gene–environment study. Journal of Youth and Adolescence. (2018) 47: 2468–2480. https://doi.org/10.1007/s10964-018-0925-3
3. Cao YM, Lin XN, Ji LQ, Zhang YP, Zhang WX. The interaction between val158Met polymorphism in the COMT gene and peer relationship on adolescents' depression. Psychological Development and Education. (2017) 33:216–227.
4. Cao C, Wang MP, Ji LQ, Wei X, Cao YM, Zhang WX. The MAOA rs6323 polymorphism interacts with maternal supportive parenting in predicting adolescent depression: Testing the diathesis-stress and differential susceptibility hypotheses. Acta Psychologica Sinica. (2016) 48: 22–35.
5. Chen XY. Analysis of the current situation and causes of mental health of secondary school students in Kunming. Master dissertation, Yunnan Normal University (2003).
6. Cheng G, Liu JQ, Lin N, Huang JJ, Wang XQ. The relationship between family socioeconomic status and mental health of secondary school students: The mediating role of psychological quality. Journal of Southwest University (Social Sciences Edition). (2019) 45:105–112.
7. Cheng J, Wang RC, Yin X, Fu L, Liu ZK. U-shaped relationship between years of residence and negative mental health outcomes among rural-to-urban children in migrant schools in Beijing, China: The moderating effects of socioeconomic factors. Frontiers in Public Health. (2017) 5:168. https://doi.org/10.3389/fpubh.2017.00168
8. Chi XL, Becker B, Yu Q, Hossain MM, Lin JY, Yeung A, Seiler-Ramadas R, Grabovac I, Bu H, Xie F, Zou LY. Persistence and remission of depressive symptoms and psycho-social correlates in Chinese early adolescents. BMC Psychiatry. (2020) 20:1-11. https://doi.org/10.1186/s12888-020-02808-5

9. Fan F. Psychopathology of anxiety and depression of left-behind children and empirical study of resilience development program. Doctoral dissertation, Central South University (2008).

10. Feng XH, Zhang XK. Researches about the relationships among pride, explicit self-esteem and depression in urban poverty adolescents. Psychological Development and Education. (2008) :100–105.

11. Guo HY, Chen LH, Ye Z, Pan J, Lin DH. Characteristics of peer victimization and the bidirectional relationship between peer victimization and internalizing problems among rural-to-urban migrant children in China: A longitudinal study. Acta Psychologica Sinica. (2017) 49:336–348.

12. Hao Z. Family socio-economic status, education expectation, parent-child communication, and child development. Youth Studies. (2013) :11-26+94.

13. Hong X, Li JQ, Liang YQ, Wang ZY, Yang HF, Qi SX, Chen XP, Xu F. Follow-up study on relationship between parental educational attainment and depressive symptoms among high school students in Nanjing. Chinese Journal of School Health. (2012) 33:697–699.

14. Hong X, Li JQ, Wang ZY, Liang YQ, Yang HF, Xu F. Study on the prevalence of depressive symptom and the association of parental occupational status with depression among high school students in Nanjing. Journal of Hygiene Research. (2013) 42:637–641.

15. Hong X, Li JQ, Xu F, Tse LA, Liang, Y Q, Wang ZY, Yu IT, Griffiths S. Physical activity inversely associated with the presence of depression among urban adolescents in regional China. BMC Public Health. (2009) 9:1-9. https://doi.org/10.1186/1471-2458-9-148

16. Hong X, Liang YQ, Wang ZY, Yang HF, Qi SX, Chen XP, Xu F. Relationship between familial socioeconomic status and depressive symptoms among high school students. Chinese Journal of Public Health. (2012) 28:473–476.

17. Huang SL, Han MY, Sun LL, Zhang HC, Li HJ. Family socioeconomic status and emotional adaptation among rural-to-urban migrant adolescents in China: The moderating roles of adolescent's resilience and parental positive emotion. International Journal of Psychology. (2018) 54:573-581. https://doi.org/10.1002/ijop.12499

18. Huo LH. A correlation research between parental rearing attitude and psychological health of vocational students. Master dissertation, Hebei Normal University (2014).

19. Ji D, Chen H, Chao M, Li XY. Effect of family atmosphere and parental education level on depression among adolescents Chinese. Journal of Public Health. (2018) 34:38–41.

20. Lai XF, Bao ZZ, Wang YH. Meaning in life and depression among adolescents: The mediating effect of self-esteem. Psychological Research. (2016) 9:28–34.
21. Liang SY. The effect of family socioeconomic status on senior high school students' mental health: The moderating effect of the subjective socioeconomic status and social power. Master Dissertation, Southwest University (2015).
22. Li DP, He D, Chen W, Bao ZZ, Wang YH, Zhao LY. School climate and adolescent problem behaviors: The mediating role of peer victimization. Journal of Psychological Science. (2015) 38:896–904.
23. Li HX. A study on the mental health status of high school students in Zhongshan City--A case study of X Middle School. Master Dissertation, Qinghai Normal University (2016).
24. Li Y, Li DP, Li X, Zhou YY, Sun WQ, Wang YH, Li JF. Cyber victimization and adolescent depression: The mediating role of psychological insecurity and the moderating role of perceived social support. Children and Youth Services Review. (2018) 94:10–19. https://doi.org/10.1016/j.childyouth.2018.09.027
25. Li S. A survey on the family environment of "left-behind children" in rural junior high schools and its impact on their mental health and academic performance. Master Dissertation, Zhengzhou University (2006).
26. Liu D. A study on depression status of high school students and its influencing factors. Master Dissertation, Shandong University (2006).
27. Liu PP, Hong W, Niu LH. A current situation survey and influence factors of adolescent depression in suburban district. Chinese Journal of Clinical Psychology. (2012) 20:668-669+742.
28. Liu SR, Miao RJ. Depression symptoms and influencing factors of primary and middle school students in Shihezi City of Xinjiang. Occupation and Health. (2018) 34:1256–1261.
29. Liu SY. Depression and its influencing factors of migrant children: A Shanghai-Based Questionnaire Survey. Master Dissertation, East China University of Science and Technology (2013).
30. Liu Y, Wang Y. Yu S. Marital Conflict, Family Socioeconomic Status, and Depressive Symptoms in Migrant Children: A Moderating Mediational Model. Behavioral Sciences, (2023) 13:441–441. https://doi.org/10.3390/bs13060441
31. Luo YZ, Wang X, Zhu XZ, Yao SQ. Levels of depression symptoms and the risk factors of anxiety in high school students. Chinese Journal of Clinical Psychology. (2008) 16:274–277.

32. Luo YM, Chen QL, Guan P, Yan J, Sun F, Han L, Jing HQ. Prevalence and influence factors of depression among 10-15 years old children in China. Chinese Journal of Public Health. (2016) 32:424–427.
33. Mao ZH, Zhao XD. The effects of social connections on self-rated physical and mental health among internal migrant and local adolescents in Shanghai, China. BMC Public Health. (2012) 12:1-9. https://doi.org/10.1186/1471-2458-12-97
34. Pan Y, Zhou D, Shek DTL. After-School Extracurricular Activities Participation and Depressive Symptoms in Chinese Early Adolescents: Moderating Effect of Gender and Family Economic Status. International Journal of Environmental Research and Public Health. (2022) 19:4231. https://doi.org/10.3390/ijerph19074231
35. Pan YG, Zhang DJ, Liu YL, Ran GM, Teng ZJ. Different effects of paternal and maternal attachment on psychological health among Chinese secondary school students. Journal of Child and Family Studies. (2016) 25:2998–3008. https://doi.org/10.1007/s10826-016-0463-0
36. Pan YY, Zhang XH. The relationship among anxiety, depression and family environment of high school students. Journal of International Psychiatry. (2017) 44:98–101.
37. Peng XJ. A study on the relationship between family environment and mental health of Wei and Han junior middle school students. Master's Dissertation, Sichuan Normal University (2008).
38. Qi SX, Hong X, Wang ZY, Yang HF, Chen XP, Zhou HR, Xu F. The effects of parents' socio-economic status on depressive symptoms of students in middle or high schools. Chinese Journal of Prevention and Control of Chronic. (2013) 21:409–411.
39. Ren HY. A comparison on the mental health of normal and professional senior high school students in Jinan city graduate student. Master's Dissertation, Shandong University (2007).
40. Sun KX. Family socioeconomic status and adolescent depressive symptoms: the mediation effect of negative life events and its gender difference. Psychological Monthly. (2021) 16:15-17+113. https://doi.org/10.19738/j.cnki.psy.2021.24.004
41. Tan YX, He T, Ding Y, Cao ZT, Lin XY. The relationship between socioeconomic status and child depressive symptoms: Analysis of multiple mediation effect. Chinese Journal of Clinical Psychology. (2023) 31:136-141. https://doi.org/10.16128/j.cnki.1005-3611.2023.01.025
42. Tang XL. A Study on the Mental Health Level of Middle School Students and its Influencing Factors. Master's Dissertation, Soochow University (2005).

43. Wang TZ, Chen MC, Sun YH, Yang LS, Sun Y, Cao QQ, Gu CM, Han TW. Research on children's depression and the influence of left-behind status in rural area. Chinese Journal of School Health. (2011) 32:1445–1446.
44. Wang LF, Feng ZZ, Yang GY, Yang YL, Dai Q, Hu CB, Liu KY, Guang Y, Zhang R, Xia F, Zhao MX. The epidemiological characteristics of depressive symptoms in the left-behind children and adolescents of Chongqing in China. Journal of Affective Disorders. (2015) 177:36–41. https://doi.org/10.1016/j.jad.2015.01.002
45. Wang LF, Feng ZZ, Yang GY, Yang YY, Wang KF, Dai Q, Zhao MX, Hu CB, Zhang R, Liu KY, Guang Y, Xia F. Depressive symptoms among children and adolescents in western china: An epidemiological survey of prevalence and correlates. Psychiatry Research. (2016) 246:267-274. https://doi.org/10.1016/j.psychres.2016.09.050
46. Wang ZH, Jin G, Lin XY. The effect of migrant children's resilience on their depression and loneliness. Chinese Journal of Special Education. (2014) :54-59.
47. Wang Q, Lin YM, Lu JK, Wu XY, Xu HL. Current status of depression symptoms and factors affecting left-behind children in northeastern Jiangxi. Chinese Journal of School Health. (2020) 41:1241-1243.
48. Wang YY, Liu ZN, Wang DF, Yang J, Zhang W, Pu WD, Zhang JQ. Psychotic-like experiences and associated factors among poor adolescents. Chinese Journal of Clinical Psychology. (2020) 28:902-905.
49. Wang SC. The effects of early trauma and recent negative life events on depressive symptoms in adolescents. Master's Dissertation, Huazhong University of Science and Technology (2019).
50. Wang YT, Yang Y, You LF. The effect of children's physical health status on depression in children. Maternal and Child Health Care of China. (2019) 34:457-459.
51. Wang WZ, Xu SS, Liu ZK, Ou Y, Gao WB. A research on the mental health of migrant laborers' children. Chinese Journal of Behavioral Medicine and Brain Science. (2007) 16:625-627.
52. Wei DP, Ma YM. A survey and analysis of the mental health status of 1848 secondary school students. Journal of Applied Preventive Medicine. (2011) 17:36-38.
53. Wu HY, Liu Y. A study on the mental health condition of students in the second senior high school in Rikaze. Tibet Education. (2011) :52-58.

54. Wu HN, Shao F, Lin B. Factors influencing the level of depression in adolescents and preventive measures. Modern Practical Medicine. (2017) 29:670-672.
55. Xiong M, Hu Z. Relative deprivation and depressive symptoms among Chinese migrant children: The impacts of self-esteem and belief in a just world. Frontiers in Public Health. (2022) :10. https://doi.org/10.3389/fpubh.2022.1008370
56. Xu FZ, Cui W, Xing TT, Parkinson M. Family socioeconomic status and adolescent depressive symptoms in a Chinese low- and middle-income sample: The indirect effects of maternal care and adolescent sense of coherence. Frontiers in Psychology. (2019) 10:819. https://doi.org/10.3389/fpsyg.2019.00819
57. Yang YJ, Tao FB, Wan YH. Depressive symptoms and the influencing factors among left-behind children. Chinese Journal of School Health. (2010) 31:321-323.
58. Yang YQ, Chen L, Chen GH, Zhang WX. Peer rejection, friendship support and adolescent depressive symptoms: A mediated moderation model. Chinese Journal of Clinical Psychology. (2020) 28:348-353.
59. Yang YQ, Chen L, Ji LQ, Zhang WX. The intergenerational transmission of maternal depression: The mediating role of mother-adolescent relationship and the moderating role of adolescents' negative affectivity. Psychological Development and Education. (2017) 33:368-377.
60. Yin XC, Li CK, Jiang SS. The mediating effect of self-esteem on the relationship of living environment, anxiety, and depression of underprivileged children: A path analysis in Chinese context. Journal of Health Psychology. (2017) 25:941-952. https://doi.org/10.1177/1359105317739966
61. Yuan XJ, Fang XY, Liu Y, Li ZR. Educational settings and city adaptation of migrant children. Journal of Beijing Normal University (Social Sciences). (2009) :25–32.
62. Yuan Y, Zhou A, Kang M. Family Socioeconomic Status and Adolescent Mental Health Problems during the COVID-19 Pandemic: The Mediating Roles of Trait Mindfulness and Perceived Stress. International Journal of Environmental Research and Public Health. (2023) 20:1625. https://doi.org/10.3390/ijerph20021625
63. Zeng RH. Analysis on the status quo and influencing factors of depression and anxiety among middle school students. China Modern Doctor. (2010) 48:86–87.

64. Zhao GM. The relationship between middle school students' depression and family socioeconomic status and mental resilience. Master Dissertation, Guizhou Normal University (2020).
65. Zhao JX. Left-home-kids' depression and antisocial behavior in the stressful context: The role of protective factors. Doctoral dissertation, Beijing Normal University (2007).
66. Zhang GX. The study on the relationship among the parental upbringing style, self-acceptance and mental health of vocational school. Master Dissertation, Hebei Normal University (2010).
67. Zhang HY, Wang J, Li XY, Chen H, Wang YC. Regulating effects of parents' educational level on the influence of character strengths of adolescent depressive symptoms. Chinese General Practice. (2016) 19:2975–2981.
68. Zhang XY. Parenting practice and its relationship with children's psychological adjustment in different socioeconomic status. Master Dissertation, Shanghai Normal University (2016).
69. Zhong B, Chen J. Health information helps mitigate adolescent depression: A multivariate analysis of the links between health information use and depression management. Child: Care, Health and Development. (2020) 47:201–207. https://doi.org/10.1111/cch.12831
70. Zhou AH. Depressive symptoms and relative factors of left-behind children from Yangcheng area of Jiangsu province. China Journal of Health Psychology. (2016) 24:1753–1755.
71. Zhou Q, Fan LB, Yin ZC. Association between family socioeconomic status and depressive symptoms among Chinese adolescents: Evidence from a national household survey. Psychiatry Research. (2018) 259:81–88. https://doi.org/10.1016/j.psychres.2017.09.072
72. Zhou YJ, Meng SS. The relationship between family economic status and depression and its implication for ideological education. Time Report. (2019) :242–243.

2 Appendix B – Study Quality Criteria and Equations for Effect Sizes

2.1 Study quality criteria:

We coded the study quality according to the following 10 criteria:

1) depression measured by standard methods;

2) reporting bias;

3) appropriate statistics used;

4) participation rate greater than 80%;

5) *N* greater than 1000;

6) randomly selected;

7) participants' other characteristics (*region*, *gender*, *age*, *etc*);

8) SES measured by two or more indicators;

9) SES reported by whom;

10) control variables.

2.2 Equations used in calculating the effect sizes:

- *Eq. (B.1)*: $r = \sqrt{\frac{F \times df_1}{F \times df_1 + df_2}}$ (*Equation for converting F value to r value*)
- *Eq. (B.2)*: $Z = 0.5 \times \ln\left(\frac{1+r}{1-r}\right)$
- *Eq. (B.3)*: $V_z = \frac{1}{n-3}$
- *Eq. (B.4)*: $r = \frac{e^{2z}-1}{e^{2z}+1}$
- *Eq. (B.5)*: $r = \sqrt{\frac{t^2}{t^2+df}}$ (*Equation for converting t value to r value*)
- For the conversion of χ^2 and β values to *r* value, please refer to:

"Lenhard, W. & Lenhard, A. (2022). Computation of effect sizes. Retrieved from: https://www.psychometrica.de/effect_size.html. *Psychometrica*. DOI: 10.13140/RG.2.2.17823.92329"

- For the conversion of *OR* value to *r* value, please refer to:

"Hamling, J., Lee, P., Weitkunat, R., & Ambühl, M. (2008). Facilitating meta-analyses by deriving relative effect and precision estimates for alternative comparisons from a set of estimates presented by exposure level or disease category. *Statistics in medicine*, *27*(7), 954-970. DOI: 10.1002/sim.3013"
